# Exploring the Impact of Social Media on Anxiety Among University Students in the United Kingdom: Qualitative Study

**DOI:** 10.2196/43037

**Published:** 2023-06-16

**Authors:** Ailin Anto, Rafey Omar Asif, Arunima Basu, Dylan Kanapathipillai, Haadi Salam, Rania Selim, Jahed Zaman, Andreas Benedikt Eisingerich

**Affiliations:** 1 Faculty of Medicine Imperial College London London United Kingdom; 2 Faculty of Life Sciences and Medicine Kings College London London United Kingdom; 3 Imperial College Business School Imperial College London London United Kingdom

**Keywords:** social media, anxiety, university student, university, college, student, qualitative, mental health, mental well-being, thematic analysis, stress, health care professional, humanistic, social science, undergraduate, narrative inquiry, narrative inquiry, social network, United Kingdom

## Abstract

**Background:**

The rapid surge in social media platforms has significant implications for users’ mental health, particularly anxiety. In the case of social media, the impact on mental well-being has been highlighted by multiple stakeholders as a cause for concern. However, there has been limited research into how the association between social media and anxiety arises, specifically among university students—the generation that has seen the introduction and evolution of social media, and currently lives through the medium. Extant systematic literature reviews within this area of research have not yet focused on university students or anxiety, rather predominantly investigating adolescents or generalized mental health symptoms and disorders. Furthermore, there is little to no qualitative data exploring the association between social media and anxiety among university students.

**Objective:**

The purpose of this study is to conduct a systematic literature review of the existing literature and a qualitative study that aims to develop foundational knowledge around the association of social media and anxiety among university students and enhance extant knowledge and theory.

**Methods:**

A total of 29 semistructured interviews were conducted, comprising 19 male students (65.5%) and 10 female students (34.5%) with a mean age of 21.5 years. All students were undergraduates from 6 universities across the United Kingdom, with most students studying in London (89.7%). Participants were enrolled through a homogenous purposive sampling technique via social media channels, word of mouth, and university faculties. Recruitment was suspended at the point of data saturation. Participants were eligible for the study if they were university students in the United Kingdom and users of social media.

**Results:**

Thematic analysis resulted in 8 second-order themes: 3 mediating factors that decrease anxiety levels and 5 factors that increase anxiety levels. Social media decreased anxiety through positive experiences, social connectivity, and escapism. Social media increased anxiety through stress, comparison, fear of missing out, negative experiences, and procrastination.

**Conclusions:**

This qualitative study sheds critical light on how university students perceive how social media affects their anxiety levels. Students revealed that social media did impact their anxiety levels and considered it an important factor in their mental health. Thus, it is essential to educate stakeholders, including students, university counselors, and health care professionals, about the potential impact of social media on students’ anxiety levels. Since anxiety is a multifactorial condition, pinpointing the main stressors in a person’s life, such as social media use, may help manage these patients more effectively. The current research highlights that there are also many benefits to social media, and uncovering these may help in producing more holistic management plans for anxiety, reflective of the students’ social media usage.

## Introduction

### Background

The rapid surge in social media platforms and users has substantial implications for mental health, particularly anxiety. In the case of social media, the impact on mental well-being has been highlighted by multiple stakeholders as a cause for concern, even being spotlighted by the government of the United Kingdom in the latest Mental Health and Well-being Discussion Paper [1]. However, there has been a lack of research into how the association between social media and anxiety arises, specifically among university students, a cohort that has witnessed the inception and progression of social media and predominantly engages with it.

Social media refers to a group of internet-based applications that build on the foundations of Web 2.0, allowing the creation and exchange of user-generated content [2]. There are currently 4.2 billion active social media users worldwide [3]. Global social media usage is projected to rise by 16.7% over the next 5 years, indicating it is here to stay [3]. Extant work notes social media’s critical role in people’s lives, including people becoming attached to their favorite social media app and experiencing separation distress when not able to use it [4-7]. Social media has also been highlighted as a potential force for good, for example, through the use of gamification, enhancing people’s happiness, and helping people quit smoking [8-13]. Important questions, however, remain regarding the impact of social media on university students. Consequently, its implications, both positive and negative, ought to be investigated, especially with regard to university students, of whom 76% have an account on some form of social media platform [14].

Behaviors stemming from social media use, including impaired sleep and sedentary practices, have been suggested as possible mechanisms for the rise of anxiety and symptoms of other mental health disorders [15]. University students, in particular, may fall victim to such behaviors, with it being found that up to 60% of all college students experience poor sleep quality [16]. Anxiety, defined by the National Health Service (NHS) [17] as a feeling of unease, such as worry or fear, is commonplace in adults in the 21st century, with anxiety disorders being the most prevalent form of mental illness [18]. Anxiety disorders in particular refer to general anxiety disorder, panic disorder with or without agoraphobia, social anxiety disorder, specific phobias, and separation anxiety disorder [19]. The importance of understanding the impact of social media on anxiety in university students is a priority now more than ever due to the increasing prevalence of both social media use and anxiety [3]. Studies within child and adolescent populations have demonstrated a significant association between social media use and anxiety [15,20]. However, this association is complicated by the various pathways outlined in much of the adolescent literature. One study described how different metrics of social media activity, including the user’s number of social media accounts and frequency of social media checking, were significantly correlated with higher levels of anxiety [21]. Another study demonstrated the implication of sleep within this relationship: increased nighttime-specific social media use resulted in later bedtimes and poor sleep, which ultimately led to increased anxiety [20]. Other phenomena have also been implicated within this relationship, including time spent, addiction, and emotional investment [15,22].

While this relationship has been well researched within child and adolescent populations, there has been limited focus on other groups, such as university students. Adolescents are at a unique stage of development and vulnerable to environmental insults such as social media use due to increased central nervous system plasticity, biological changes, and the shaping of psychological mechanisms [23]. Yet, university students are the population most susceptible to mental health disorders, with anxiety prevalence higher than the general population average at 11.2% [24-27]. Researchers often consider university students as a distinct population, also with their own unique set of risk factors that have been proposed as contributing toward the anxiety reported. These include strenuous academic demands, challenges of leaving home, distance from support networks, and a harmful predisposition to substance abuse, all specific to this cohort [28,29]. These significant differences in environmental context may affect the interactions these groups have with social media and the resultant impact on anxiety, thus limiting the generalizability of existing adolescent population findings within university student cohorts. Therefore, to build on the limited literature focused on the university student population, a systematic literature review (SLR) on the impact of social media on anxiety was conducted.

### Systematic Literature Review

**Figure 1:**
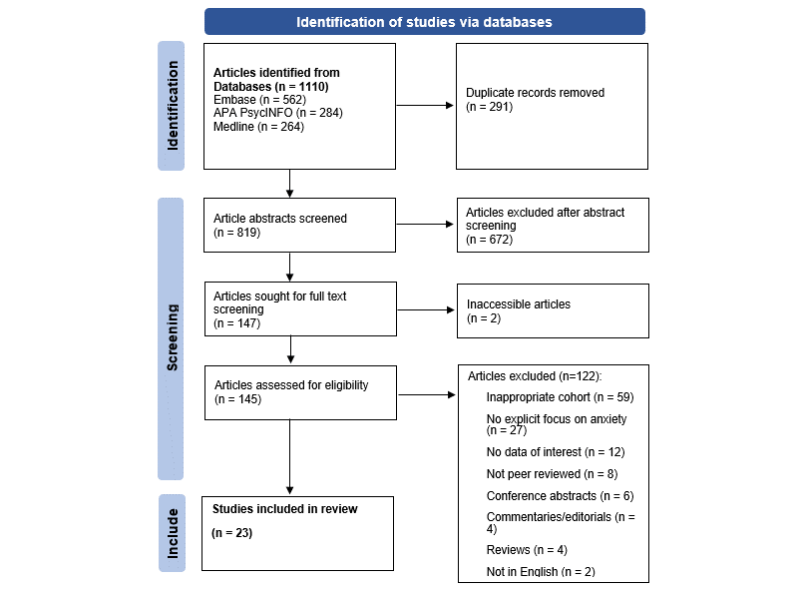
PRISMA (Preferred Reporting Items for Systematic Reviews and Meta-Analyses) flow diagram.

The protocol for the systematic review was registered with the International Prospective Register of Systematic Reviews (Prospero; CRD42022304959) and conducted in accordance with guidelines from the PRISMA (Preferred Reporting of Items for Systematic Reviews and Meta-Analyses) statement [30]. A systematic search of the literature was conducted in 3 electronic databases: OVID, MEDLINE, Embase, and PsychINFO, from the inception of the databases up to December 13, 2021, as shown in Figure 1. Search terms were identified prior to the search to generate the search strategy (Multimedia Appendix 1 [31-53]). A total of 23 studies (Multimedia Appendix 1) were included for analysis within this review from the 1110 available papers following abstract and full-text screenings according to the inclusion and exclusion criteria by 3 independent researchers (Multimedia Appendix 1). For each included study, researchers extracted the author names, country of origin, study design, sample cohort, sample demographic data, relevant exposure measures, relevant outcome measures, and main findings (Multimedia Appendix 1). Owing to the diverse characteristics of the selected studies (ie, study designs, intervention types, or outcomes), a narrative synthesis approach was adopted for a more transparent presentation of findings.

### Principal Findings

This systematic review explored the current literature on the association between social media use and anxiety among university students, and it was evident that the association is multifaceted. The included studies were generally of fair to good quality, thus suggesting reasonable validity in our findings.

This review identified that the most commonly investigated factor impacting levels of anxiety was the frequency of social media use, with contrasting associations across the studies [31-35]. Only 1 paper demonstrated a significantly positive association, with increased Facebook use leading to higher anxiety levels [31]. The remaining studies, however, highlighted significant relationships across other components of social media use beyond the frequency of use.

The nature of social media activity that university students engaged in was a more significant component in evoking anxiety. It was found that the viewing of certain image types induced higher levels of anxiety. Specifically, anxiety levels were immediately higher following the exposure of students to thin-ideal images, with anxiety-reducing as time elapsed [36]. Another study shared similar findings, with anxiety increasing following activity involving exposure to appearance-focused Instagram accounts [37]. This may be explained by social comparison theory, whereby comparison of one’s body to others may threaten one’s self-evaluation leading to feelings of anxiety [54]. Interestingly, while this behavior is more commonly observed in adolescents, our findings suggest that this phenomenon is present in university students as well [55,56]. This is further supported by another study identifying envy as a potential mediator between the relationship between social media and anxiety [38].

Mediators are indirect pathways through which social media use impacts anxiety levels. Further to envy, another key mediator identified as stress, with social media use increasing stress levels and, consequently, anxiety levels. The positive association between stress and anxiety levels has previously been highlighted in prior literature [56,57], with potential biochemical changes in the body providing an explanation for this association. The role of social media in exacerbating stress has also been previously identified within adolescents [58] through feeling overwhelmed as well as through indirect ways such as impacting sleep [59]. Psychological capital was another mediator identified, referring to a combination of positive psychological constructs including hope, resilience, optimism, and self-efficacy [60]. Social media use had a significant negative effect on psychological capital, while psychological capital decreased anxiety levels. This finding may be particularly useful in attempting to moderate anxiety levels in this population.

Additionally, a few experimental studies showed how social media abstinence can be beneficial in reducing anxiety levels, which is an important consideration in identifying techniques to aid those that experience increased anxiety levels as a result of social media use [39,40].

### Conclusion and Design of the Primary Study

The SLR demonstrated that various elements and characteristics of social media can impact anxiety beyond the frequency of use. The SLR further identified a significant lack of qualitative studies within this field of research, highlighting the inadequate depth of exploration on the subject. Further studies are needed to address the underlying pathways and mechanisms through which social media impacts anxiety levels within this population. Additionally, there was a distinct lack of studies that investigated this association in the United Kingdom.

### Aims and Objectives

We conducted a qualitative study to examine the impact of social media on university student’s anxiety levels through qualitative research.

## Methods

### Overview

Primary data collection was carried out through a mono-method qualitative study approach, in line with an interpretive and inductive approach to underpin and reinforce existing theory and carefully navigate the complex and subjective nature of the interactions between social media and anxiety perceptions [61]. This study was conducted as per ethical approval by the Imperial College Research Ethics Committee, following assessment by the Research Governance and Integrity Team and Head of Department on February 8, 2022 (ICREC reference number: 21IC7395).

### Setting and Sample

A total of 29 semistructured interviews were conducted, comprising 19 male students (65.5%) and 10 female students (34.5%) with a mean age of 21.5 years. All students were undergraduates from 6 universities across the United Kingdom, with most students studying in London (89.7%). Participants were also asked to self-report their ethnicity using the UK Office for National Statistics ethnicity categories. Participants were enrolled through a homogenous purposive sampling technique via social media channels, word of mouth, and across different university faculties in different regions of the United Kingdom. Recruitment was suspended at the point of data saturation. Participants were eligible for the study if they were university students in the United Kingdom and users of social media.

### Data Collection

An exploratory pilot interview was conducted prior to data collection, allowing for analysis regarding length, style of question, and interviewer mannerisms to ensure consistency amongst interviewers [62-64].

The semistructured interview guide (Textbox 1) was informed by the aims and objectives of the project, the systematic review findings, and the pilot interview learnings. The interview guide focused on eliciting views and feelings involving (1) the participants’ current social media use, (2) the participants’ general opinions on social media, and (3) the participants’ perceptions of the impact of social media on their feelings of anxiety.

Web-based interviews were conducted via Microsoft Teams and Zoom between March and April 2022. Interviews consisted of 2 interviewers: 1 principal interviewer and 1 scribe. Interviews lasted approximately 30-60 minutes and were transcribed.

**Semistructured interview guide for face-to-face individual interviews.**

- Social media usage
  Describe how you use social media at the moment. (Clarify using probing questions below if necessary)
  Which social media platform do you use? 
  How much of each social media platform do you use each day? 
  When do you use social media the most?
  What level of activity do you have on social media (passive or active)? For example, passive would be you just scrolling through, and active would be where you are interacting with posts such as messaging, sharing, posting, commenting, and so on.
  What does your use of social media consist of?
  What type of content do you view using social media?
  What are your motivations for using social media?
- Social media perceptions
  What are your views on social media?
- Impact of social media on mental health
  Has social media usage impacted your well-being? If so, how?
  Has social media usage impacted your mental health? If so, how?
  Has social media ever explicitly benefited you? If so, how?
  Has social media use explicitly upset you? If so, how?
  Has social media impacted your anxiety on a day-to-day basis? If so, how?
  Has social media ever impacted your anxiety symptoms? If so, how?
  Which social media platforms have the biggest impact on your anxiety? Are there any reasons for this?
  What type of content or media has the biggest impact on your anxiety?

### Data Analysis

#### Overview

Thematic analysis of the semistructured interviews was conducted using Braun and Clarke’s 6 steps to provide a rich yet complex analysis of the data in a flexible and robust manner [61,65]. Gioia et al’s [66] approach was used to construct the data structure to ensure a rigorous inductive research approach. A concurrent and recursive analytical approach was used, in which researchers analyzed data during collection and coding to refine codes and search for themes. To achieve consensual validation of any discrepancies, 3 researchers carried out a thematic analysis.

#### Phase 1: Data Familiarization

Each investigator read all the transcripts independently, generating concepts, and capturing ideas that explain the phenomena of interest.

#### Phase 2: Generation of First-Order Codes

First-order codes were generated independently by each investigator to link data referring to the same specific meanings [61]. Researchers then discussed the first-order codes identified and combined similar ideas under a single code, conscientiously adhering to participants’ terms.

#### Phase 3: Generation of Second-Order Themes and Subthemes

Investigators sought out similarities and differences among the first-order codes, categorizing these into subthemes and then into second-order themes. To produce a vibrant inductive model, researchers acknowledged the interrelationships among emergent themes. This formed the data structure, which visually demonstrates the process by which raw data transforms into second-order themes.

#### Phases 4 and 5: Defining, Naming, and Review of Themes

Themes and their encompassing components were reviewed, refined, and reorganized in a 2-level process by the 3 researchers. First-order codes were reviewed to test for coherence and relevance in their overarching subtheme. The subthemes and second-order themes were then refined to ensure relevance to research aims and an accurate representation of the data. The entire data set was revisited, and additional data were recoded that may have been missed in earlier phases. The names of the themes were crafted to be clear and instructive.

#### Phase 6: Producing a Report

Findings were presented through an analytic narrative approach that included explanations for each theme accompanied by examples from participants to demonstrate the merit and validity of the analysis [65,66].

### Research Trustworthiness

The 4 criteria by which Guba [67] establishes trustworthiness are credibility, transferability, dependability, and confirmability [68]. Credibility was ensured through member checking of transcriptions, whereby participants were provided with their interview transcripts to guarantee accuracy [61]. To establish transferability, demographic characteristics have been provided for all participants [61,68]. Dependability was achieved through auditing the methodology, as demonstrated by the pilot interview [69]. To ensure confirmability, this study’s methodology and rationale served as a rudimentary audit trail, and stages such as thematic analysis were conducted independently by multiple researchers.

### Ethics Approval

Ethical approval was given by the Imperial College Research Ethics Committee (ICREC reference number: 21IC7395). This study followed the principles of the Helsinki Declaration and the Danish Code for Research Integrity. Informed consent was obtained verbally and in writing from all participants, and they had the right to withdraw from the study at any given point. The interviews conducted were private, and the study data was kept anonymous, including from other members of the research team. Additionally, participants did not receive any compensation for their participation.

## Results

### Overview

A total of 29 student interviews were conducted; Table 1 represents a summary of the demographics of the participants interviewed.

Student participants’ experiences and perceptions of social media use and its effect on anxiety were analyzed. The analysis resulted in 8 second-order themes: 3 factors that decrease anxiety levels and 6 factors that increase anxiety levels (Textboxes 2 and 3). Factors perceived to be decreasing student anxiety levels were social connections, positive experiences, and escapism (themes 1 to 3, respectively). Factors that were perceived to increase student anxiety levels were comparison, fear of missing out (FOMO), procrastination, stress, and negative experience (themes 4 to 8, respectively), summarized.

These second-order themes are further organized into subthemes, which are elaborated on in detail, providing quotes from participants for further harmonization and understanding. First-order concepts and selected quotes illustrate these themes (see Multimedia Appendix 2). We have replaced participant names with participant numbers (eg, Student 1).

**Table 1:** Demographics of interview participants (N=29).

Characteristics			Participants, n (%)
**Gender**			
	Male	19 (65.5)	
	Female	10 (34.5)	
**Age (years)**			
	18-20	3 (10.3)	
	21-23	26 (89.7)	
**Ethnicity**			
	White	3 (10.3)	
	Asian or Asian British	23 (79.3)	
	Black, African, Caribbean, or Black British	1(3.45)	
	Other	2 (6.9)

**Second-order themes and subthemes of factors decreasing student anxiety.**

**Social connections**

- Connect with friends and family
- Communities

**Escapism**

- Distraction
- Relief

**Positive experiences**

- Positive validation
- Positive content

**Second-order themes and subthemes of factors increasing student anxiety.**

- Comparison
  Comparison of life
  Comparison of body type
- Fear of missing out
- Procrastination
- Stress
  Feeling overwhelmed
  Overthinking interactions
- Negative experiences
  Disturbing and harmful content
  Cyberbullying
  Judgment by others

### Theme 1: Social Connections

#### Subtheme 1.1: Connection With Friends and Family

A majority of participants (n=19) spoke of the benefits of social media as a tool to create and maintain social connections. When students were worried about long-distance relationships, social media platforms allowed them to maintain these relationships, thus overcoming geographical barriers.

I’m a very social person so I like that side of social media. It helps me stay connected with people all around the world, even my cousins who live abroad

Student 22

Social media provided students with a unique opportunity to grow their social circles by building and even reestablishing friendships:

I’ve met like some of my closest most dear friends through it

Student 7

I lost contact with him for 10 plus years. We found each other through Instagram. So, we started messaging again.

Student 12

Importantly, social media prevented student loneliness and social isolation during difficult times during their transition into university or during the COVID-19 pandemic when students were quarantined:

In terms of my mental well-being, it can help me stop loneliness and keep in touch with friends and family especially when I’m at university.

Student 27

So even though you’re at home, by yourself, or with just your family, you’re actually like, connected with the world out there.

Student 6

#### Subtheme 1.2: Web-Based Communities and Support

The establishment of connections through social media has offered opportunities for students to surround themselves with supportive web-based communities of like-minded people. These have formed sources of support and praise:

I’m fortunate enough to belong in one where everyone seeks to uplift one another, with the work you are producing being praised and promoted.

Student 21

In lockdown, where a lot of people were then doing like the virtual calls and virtual iftars and virtual like, talks and things and it created such a nice community.

Student 6

### Theme 2: Positive Experiences Associated With Social Media Usage

Positive experiences are described as social media interactions that induce happiness and inspiration in students, especially when they are in a low mood. This encompassed viewing and interacting with positive content and receiving positive validation.

#### Subtheme 2.1: Positive Content

There were plentiful types of positive media consumed by students (n=10) on social media, including spiritual, creative, informational, and activist content. This motivated students to adopt healthier lifestyles and inspired creativity.

I can follow someone that has a healthy living account for example I find that seeing posts like that would encourage me to be better or to go out and go for a run or pick something healthy to do.

Student 15

So in terms of creativity, it makes me happy seeing other people’s work and then using that to inspire myself.

Student 17

Students revealed that social media enables greater visibility and support for social issues and activism. Through awareness and education on global movements through social media, students bonded with others and expressed solidarity.

I’ve learned a lot from people through social media, just different people’s mindsets and different people’s viewpoints. I think specially with certain campaigns, such as BLM [Black Lives Matter] for example, that really showed the power of people when they come together.

Student 6

Another benefit mentioned by some students (n=3) was access to religious content, which induced a sense of hope and motivation.

Social media has benefited me a great deal in terms of coming closer to my own faith. I follow a lot of religious accounts on social media and they tend to show positive, bitesize clips that really provides me with hope and motivation to continue being strong in your faith.

Student 27

#### Subtheme 2.2: Positive Validation

Some students mentioned that social media was a channel to receive positive validation and compliments (n=5). Students revealed that they were positively impacted by web-based exchanges with peers:

The endorphins that come with posting or the number of likes does have a positive impact on me

Student 22

It’s nice getting positive feedback from people on stuff that you posted and it’s nice receiving compliments, but also complementing other people on stuff they’ve posted.

Student 15

### Theme 3: Escapism

Social media was described as providing students with an escape from mental health issues or any other stressors that may be plaguing their thoughts, thereby providing them with a sense of calm and relief.

#### Subtheme 3.1: Distraction

To mentally cope with and divert feelings of anxiety, students discussed using social media as an instrument to distract from or avoid their problems.

If there is anything in the real world that affects my anxiety, funnily social media can act as a way of helping me cope with it. It puts my mind in another place; a place where I don’t have to think too much

Student 22

Pull me out of my like, state of like despair, into anything that’s stimulating. That’s why I was like, watching things that were just like, utter trash, like it made no sense. But like, it was something to just distract me. And that’s what I needed at that time.

Student 4

#### Subtheme 3.2: Relief

Social media is commonly used for leisure and mindless entertainment to relax and reduce anxiety levels.

It’s also a nice break in the day so if you had a busy day at work you can just have a quick scroll through.

Student 16

Sometimes if you’re just feeling a bit you know, as you put like, a bit anxious or maybe overwhelmed. Just doing something pretty mundane, can sort of help.

Student 20

### Theme 4: Comparison

A major contributor to anxiety in students (n=22) was described as the comparisons that they would draw between their own lives and the lives of others seen on social media, be that people that they know personally or other personalities such as influencers or celebrities.

#### Subtheme 4.1: Comparison of Life

Students specifically discussed the comparison of factors that were external to their own physical being. A common thread of comparison was that of the participants’ social lives, for example, seeing how many friends others had or the enjoyment that their counterparts attained from social gatherings. Seeing such content from peers would drive students to be anxious.

When people post a lot of content about their lives whilst you’re just at home, not doing anything special, you feel anxious in that you should really be doing something. Or that I am not doing enough

Student 21

Students mentioned that they also compared their academic performances and other life achievements to others, saying that they felt anxious about not performing to the same standard:

...just looking at the success of others and comparing it to my lack of success really heightened my anxiety and pushed me down further.

Student 27

One student mentioned the comparison of wealth and social status as a reason for anxiety:

So you can actually see who is financially more well off or of a higher social economic class and then you’re kind of comparing yourself to these people who have more money than you

Student 10

#### Subtheme 4.2: Comparison of Body Types

Students felt that image-based social media platforms contributed to anxiety as they led them to compare their appearance to the perceived ideals that they saw on platforms.

I had like an issue with body image. It wasn’t a big thing, but it played on the back of my mind. And with social media like Instagram, where a big part of it is posting photos of yourself, I could definitely see why it would be a negative

Student 11

I think it comes down to influencers – like they have good body types and things that can make you quite insecure.

Student 25

### Theme 5: FOMO

The concept of FOMO was cited by students as another noteworthy outcome of social media use that contributed to anxiety. FOMO consists of the feeling that by being away from social media, one could eventually feel anxious about missing out on messages.

I would not use my phone for even just 2 hours and I would feel like I was missing out on something. This would drive me crazy and add so much unnecessary stress

Student 24

Additionally, in some cases (n=2), FOMO was mentioned to be occurring because of comparison to what other people posted on social media:

You see so many different stories of people doing different things. Sometimes you get that fear of missing out.

Student 11

People around me were always on their phones using these apps, so naturally, I would do the same. If I wasn’t on it, it would bring the fear that I felt like I was missing out

Student 24

### Theme 6: Procrastination

Students (n=13) discussed how the nature of social media is such that they would constantly return to it and hence consume a lot of time. This impulsive use caused them to use social media, thereby procrastinating and causing anxiety from the feeling that they could have been more productive with their time.

if I have a deadline or things to do, like exam preparation, I am not one to just focus on that. I tend to procrastinate an unhealthy amount. Social media takes time away from me completing my uni work, doing my chores or doing things to better myself and, in turn, this increases my anxiety levels.

Student 22

### Theme 7: Stress

Students also mentioned (n=8) how social media was a large cause of stress through various mechanisms, which in turn caused them to be more anxious.

#### Subtheme 7.1: Feeling Overwhelmed

Excessive connectivity to others through social media would lead to students feeling overwhelmed by an unmanageable number of notifications. The perceived obligation to respond rapidly was what drove students to feel anxious.

With WhatsApp, there’s kind of a chronic stress and background, especially when you have like later messages and stuff coming through and you can kind of feel obliged to kind of just continually be online and be active

Student 5

#### Subtheme 7.2: Overthinking Interactions

Constant contact with people through direct messages or posts on social media led to students overanalyzing the web-based interactions that they had. Students were self-conscious in their social media interactions, which made them anxious about how their peers might respond.

There is so much overthinking with it; thinking that maybe this person didn’t like me. Or perhaps you message someone, and you see that they are online, but they do not message you. Again, sometimes I look at this and think “Ok, does this person not have enough time for me?”

Student 27

### Theme 8: Negative Experiences

A common theme that arose from the interviews (n=18) was negative experiences on social media, which were described as those that cause displeasure and unhappiness in students. These negative experiences encompass the consumption of disturbing and harmful content, perceived judgment from others, and being a victim of cyberbullying.

#### Subtheme 8.1: Disturbing and Harmful Content

A striking factor that induced anxiety, as mentioned by students, was the consumption of distressing content. This was mentioned in multiple contexts; for example, viewing distressing media on the internet was said to cause negative emotional states, possibly even exacerbating symptoms in those already experiencing mental health disorders.

An example of such distressing content is the web-based promotion and glamorization of self-harm–related media.

I think it is scary to think that people can follow pages and accounts that may encourage them to self-harm or to commit suicide or to restrict eating or things like this

Student 15

#### Subtheme 8.2: Judgment by Others

Social media encouraged student behaviors such that they would be anxious about being judged by peers they were connected with. This potential judgment led to students feeling anxious about potential disapproval by others.

I think it mostly lies in the fear of not knowing how it will be perceived by others. I don’t think it’s an innate thing. I think it’s not knowing how other people will react to it

Student 18

Posting was an intensely anxious endeavor where students were concerned about the number of likes obtained, even considering removing a post if it did not perform well.

I think twice about keeping pictures up that didn’t receive as many likes as other posts of mine…it is embarrassing if you do not hit a certain number. It makes you feel less valued and less appreciated.

Student 27

#### Subtheme 8.3: Cyberbullying

A considerable negative experience that students had on social media was receiving web-based abuse and negative comments or remarks from others, otherwise known as cyberbullying. This phenomenon had a long-lasting negative impact on students’ anxiety due to the intensely personal nature of the comments:

There were a few people who used to DM (direct message) me. They would talk about my appearance, the way I look and because I am quite self-conscious about that, they would use it to target me and harass me.

Student 23

## Discussion

### Overview

To the best of the authors’ knowledge, this study is one of the first qualitative studies exploring the perceptions of university students on the impact of social media on their anxiety. The results from the SLR suggest that social media use was associated with greater anxiety levels among university students and identified mediating pathways through which this association may occur: stress, envy, psychological capital, and a negative emotional state. Our first qualitative analysis added to the literature by identifying other pathways. These pathways were categorized into those that decrease or increase anxiety levels.

### Principal Results

#### Theme 1: Social Connectivity

The primary study identified that social connectivity plays a role in decreasing anxiety levels among university students. This route is consistent with the pathway of positive relationships proposed by [70] and in prior literature [71]. According to the 4 drives theory [72], humans have an intrinsic need to seek and develop mutual social commitments. Thus, our findings suggest that social media creates an alternative route through which students can connect and establish meaningful bonds with friends and family, satisfying such a need. Additionally, the relevance of social connectivity to decreased anxiety levels has been well established [73,74]. Contrary to such findings, Rae and Lonborg [41] found that those seeking new connections beyond friends and family experienced greater anxiety with increased social media use.

Our qualitative research revealed that students relied on social media for social connectivity during the COVID-19 pandemic. Dos Santos et al [75] highlighted the potential association between social isolation during the COVID-19 pandemic and symptoms of anxiety. Focusing on the unique circumstances of the university student cohort, who tend to be detached from their family and friends, the importance of social connectivity on anxiety may be greater, especially for international students [76]. Web-based community and support were another way through which students noted that social media reduced their anxiety. Humans display a need to belong, with prior literature highlighting that an increased sense of belonging (or subjective group identification) was associated with decreased anxiety [77].

#### Theme 2: Positive Experience

Positive content, such as spiritual, creative, informational, and activist content, was noted by students to reduce anxiety levels. For instance, activist content mentioned included campaigns such as the “Black Lives Matter” movement. This pathway could potentially be explained by the cohesion and sense of solidarity that activism provides, building a sense of unity and reducing anxiety levels [78]. Furthermore, engaging with spiritual content can lead to decreased anxiety levels, with individuals mentioning that some spiritual content was both grounding and hope-inducing, as supported by the literature [79].

Validation was highlighted as another positive experience that students had on social media. Assuming a student’s basic needs are secure, social media has the potential to fulfill self-esteem needs, encompassing the desire for reputation or prestige [80]. Student participants corroborated this positive effect of external validation through social media by noting the positive effect that it had on their present dispositions. This pathway for the alleviation of anxiety is explained by the fact that users have been found to receive endless joy so long as they receive positive validation in the form of likes and responsive comments [81]. Thus, when it comes to social media use, positive outcomes for students are inextricably linked to the positive responses that they receive [82].

#### Theme 3: Escapism

Escapism has been defined as individuals choosing to get away from the reality that they live in due to unsatisfying life circumstances [83]. Students mentioned that sometimes social media provided them with an avenue to avoid negative emotional states such as anxiety by preoccupying themselves with distractions on social media. While escapism has generally been regarded in the literature as a negative and dysfunctional phenomenon [84], functional escapism has been found to be an effective coping mechanism to produce favorable well-being outcomes [85]. The description of social media use as a coping mechanism by students to alleviate anxiety follows the concept of self-suppression, as outlined by Stenseng et al [85]. While, in the short term, this provides students with a reprieve from their anxiety, it is only momentary and simply a tactic to minimize ill-being rather than one to maximize well-being [85]. That said, from our qualitative analyses, it was apparent that students viewed this as a virtue of social media, giving them an avenue to relax and find some relief in times when they may feel overly anxious.

#### Theme 4: Comparison

The findings of this study support social comparison theory [37], which is the idea that one’s personal worth is established by self-evaluations determined by comparison to others. It was evident that students compared themselves to others on social media in 2 main ways: comparison of life and comparison of body types. Students described numerous occasions of upward comparison through social media. This is the act of comparing oneself to others perceived as superior in certain domains, such as social life, academia, wealth, and social status. Social comparison is well established as a pathway that increases the risk of anxiety [86]. Certain social media platforms, such as Instagram and Facebook, which are heavily image-based, have been identified as the most triggering comparisons [86]. Prior research has suggested that reduced self-esteem is another intermediate outcome resulting from social comparison, which in turn leads to greater anxiety [87]. This suggests that the relationship between social comparison and anxiety is complex and could involve other pathways.

Academic comparison on social media was found to be particularly prevalent within the university student cohort, which then led to anxiety. Competition is one possible explanation for this, as is fear of judgment by others on the internet [88,89]. Charlesworth [90] distinguishes 2 types of competition: integrative and aggressive. Students in this study displayed a mix of both, expressing happiness for their peers while also feeling hostile competitiveness owing to their own lack of achievement. Research has shown that anxiety can be triggered when one feels as if they are losing or failing [91]. Competition is not a driver for social media usage [92]; however, it can be hypothesized that individuals who are more competitive may be more prone to experiencing anxiety due to academic comparison on social media.

Comparison of one’s own body type to others seen on social media was especially common on image-based social media such as Instagram. This is corroborated by research that has revealed photo-based activity on social media to be linked to body image dissatisfaction. Kohler et al [37] found that beauty and fitness content marginally increased anxiety among university students. Our qualitative research demonstrated that while some students compared their bodies to images posted by their peers, others openly compared their bodies to influencers. Comparing one’s body to that of others briefly threatens one’s self-evaluation, causing feelings of anxiety [36].

#### Theme 5: FOMO

FOMO, or the “fear of missing out,” was also identified as a pathway through which social media increased levels of anxiety symptoms. FOMO tends to arise through the perception of missing out, followed by actions to maintain these social connections [93]. Students mentioned that on social media platforms, individuals tend to share positive aspects of their lives, including group gatherings or partaking in various activities. Thus, propagating the feeling that their peers are engaging in more rewarding experiences. Drawing on the 4 drives theory [71], humans have a drive to acquire, thus fueling their need to match the actions and experiences of their peers. Our qualitative findings also identified that comparison drove the feeling of FOMO. This finding highlights the potential sequential nature of the mediating pathways, indicating that these pathways may not be linear in nature.

#### Theme 6: Procrastination

Procrastination was identified as another mediator, with increased social media use resulting in increased procrastination of tasks such as academic work or other daily activities, which in turn contributed to increased anxiety levels, corresponding with existing literature [94]. Additionally, qualitative findings outlined that procrastination on social media prevents students from completing other tasks such as exercise, a protective factor against anxiety [95].

Interestingly, the SLR exhibited mixed results for time spent on social media is associated with anxiety [30,31]. However, this may be due to students’ perceptions of their social media use; those who deem their time spent on social media as “waste” or procrastination may experience greater anxiety symptoms.

#### Theme 7: Stress

Consistent with prior literature [42], stress acted as a mediating pathway through which social media use drove anxiety levels among student participants. Our qualitative study highlights that this occurs as social media use provokes the overthinking of interactions, leaving students overwhelmed. This could be induced through excessive messages, explained by the theory of reciprocity, which results in individuals feeling obligated to reply to those who have taken the time to message them, otherwise resulting in reciprocity anxiety [96]. One of the key reasons mentioned that encouraged social media use was connectivity; however, it may be possible that a fine line exists between social media being beneficial for connections and acting as a stressor. Prior literature has identified that social media can drive stress levels, potentially through other mechanisms such as misinformation [97]. Thus, increased stress levels can drive anxiety, as explained by the increase in adverse psychological reactions caused by stress [98,99]. Students also highlighted that social media led them to overthink interactions, increasing their anxiety levels. However, overthinking interactions (or rumination) is a possible symptom of anxiety, and so overthinking interactions could be seen as an indication of heightened anxiety symptoms rather than a mediating pathway.

#### Theme 8: Negative Experiences

Our primary study defined negative experiences as encountering hostile web-based interactions in the form of cyberbullying, exposure to distressing and disturbing content, and the fear of judgment from others. Research suggests that cyberbullying and cybervictimization are prevalent in the student population [100], with students who are victims of cyberbullying being predicted to harbor higher levels of anxiety and even suicidal thoughts [100-102]. Conversely, some research has shown that anxiety predicts greater cyberbullying victimization among students, drawing a potential reverse pathway [103]. In our study, students described the fear of being judged by peers through social media. Many students spoke of the anxiety induced by posting content about themselves and the performance of that content. A qualitative study in the United Kingdom of people aged 8-20 years mirrored these concerns [104], acknowledging that the possible scrutiny from others prompted anxiety. This observation may be explained by the concept of self-presentation, defined by Leary [105] as “the process by which people convey to others that they are a certain kind of person.” Web-based self-presentation was shown to have a significant relationship with judgment anxiety [106,107]. However, among college students, strategic self-presentation is reported to be associated with increased well-being; thus, the link between self-presentation and anxiety still needs to be explored in depth [107].

### Limitations and Future Research

This study has several limitations, which offer avenues for future research. First, this study relied on SSI for data collection; therefore, findings may be affected by mono-method bias [108]. The use of other quantitative and alternative qualitative methods may have resulted in a more rigorous analysis. Second, pilot interviews were conducted to test the effectiveness of our questions. All subsequent interviews were carried out by 2 groups of researchers, which helped create consistency in communication styles between interviews. However, this may result in interviewer bias; therefore, training and discussion throughout the interview process were used to minimize this effect. Third, despite reaching theoretical saturation and disseminating our recruitment material to different universities across the United Kingdom, our student sample mainly consisted of students from British Asian ethnic backgrounds (n=19) and those studying in London (n=26). Additionally, the majority of the participants were male (n=19). While the data was collected from a pool that was not directly reflective of the university population in terms of ethnicity proportions, gender ratio, and geographical location, the responses reached theoretical saturation, so further consequential interviews no longer added any new findings. However, we acknowledge that for future research within this domain, greater efforts will need to be made to ensure that participants adequately reflect the greater population. Due to the sensitivity of the subject of the interviews, both groups of participants were provided with a comprehensive informational sheet containing the details of the research. Thus, participants may be subjected to anchoring and availability bias during interviews. To mitigate this, participants were asked neutral, open questions, followed by specific questions on how social media benefits them. An effort was made to ensure that the positive impacts of social media on anxiety were explored in as much depth as the negative impacts.

To enhance confidence in the generalizability of the current findings, we encourage future research to widen the study sample to include participants from a greater number of universities across the United Kingdom, including a wider demographic that is more representative of the population of the United Kingdom. Furthermore, throughout our interviews with students, it was identified that certain social media platforms have a greater effect on SMIA. Participants mentioned that platforms, like Instagram, impacted them more than others, thus emphasizing the need for future research and drawing comparisons and granularity with respect to individual platforms. Moreover, anxiety was chosen due to its prevalence among university students. Nevertheless, other mental health symptoms, such as depressive symptoms, are also prevalent within populations and richly deserving of future study [109]. Hence, we encourage additional research that focuses on these other mental health symptoms that are also deserving of in-depth research.

### Conclusions

This study sheds light on how university students perceive how social media affects their anxiety levels. The findings of this study can be used by many parties, including university counselors, students, and health care professionals, to create materials that will better prepare students to deal with anxious feelings that may arise from using social media.

## Appendix

Multimedia Appendix 1 Systematic literature review.

Multimedia Appendix 2 Thematic analysis.

## Abbreviations

FOMO: fear of missing out
PRISMA: Preferred Reporting Items for Systematic Reviews and Meta-Analyses
SLR: systematic literature review

## Data Availability

The data sets, including the second-order themes, subthemes, and first-order codes generated during or analyzed during this study, are available from the corresponding author upon request.
