# Supplementary material for: Exploring the Impact of Social Media on Anxiety Among University Students in the United Kingdom: Qualitative Study
Source: JMIR Form Res. 2023 Jun 16;7:e43037. doi: 10.2196/43037 (PMC10337317; doi:10.2196/43037)
Supplement: Multimedia Appendix 1 [file formative_v7i1e43037_app1.docx]

## Multimedia Appendix 1 - Exploring the Impact of Social Media on Anxiety Among University Students in the United Kingdom: A Qualitative Study

This is a Multimedia Appendix to a full manuscript published in the J Med Internet Res. For full copyright and citation information see http://dx.doi.org/10.2196/jmir.xxxx

### Multimedia Appendix 1: Systematic Literature Review

A.1: Search strategy

| **Social Media** | social media, online social network*, media sharing site*,  facebook, linkedin, twitter, instagram, youtube, myspace, snapchat, tiktok, reddit, whatsapp |
| --- | --- |
| **Anxiety** | anxious*, anxiet*, nervousness |
| **Students** | student*, undergraduate*, universit*, college, postgraduate* |

A.2: Search Terms used

| **Medical Subject Headings** |
| --- |
| Social Media, Online Social Networks, Anxiety, Anxiety Disorders (exploded: generalised anxiety disorder, social anxiety), Performance Anxiety |

A.3: Inclusion and Exclusion criteria

| **Inclusion Criteria** | **Exclusion Criteria** |
| --- | --- |
| University students who use social media | Papers concerning children (<18 years) |
| Mean age between 18-29 years | Papers that are not within the definition of social media (for e.g. internet addiction) |
| Social media measured as an exposure and anxiety measured as an outcome | Excluded study types: systematic reviews, scoping reviews, editorials/opinion pieces/commentaries, book chapters, case reports, clinical studies, theoretical studies, intervention studies, treatment studies. |
| Accepted study types: cohort studies, cross-sectional studies, qualitative studies, whatever is relevant | Non peer-reviewed papers |
| Papers published in English | Social media only mentioned as a moderating factor |
| Peer reviewed | Papers looking at the impact of anxiety on social media usage |

A.4: Risk of Bias Assessment

| **Inclusion Criteria** | **Exclusion Criteria** |
| --- | --- |
| University students who use social media | Papers concerning children (<18 years) |
| Mean age between 18-29 years | Papers that are not within the definition of social media (for e.g. internet addiction) |
| Social media measured as an exposure and anxiety measured as an outcome | Excluded study types: systematic reviews, scoping reviews, editorials/opinion pieces/commentaries, book chapters, case reports, clinical studies, theoretical studies, intervention studies, treatment studies. |
| Accepted study types: cohort studies, cross-sectional studies, qualitative studies, whatever is relevant | Non peer-reviewed papers |
| Papers published in English | Social media only mentioned as a moderating factor |
| Peer reviewed | Papers looking at the impact of anxiety on social media usage |


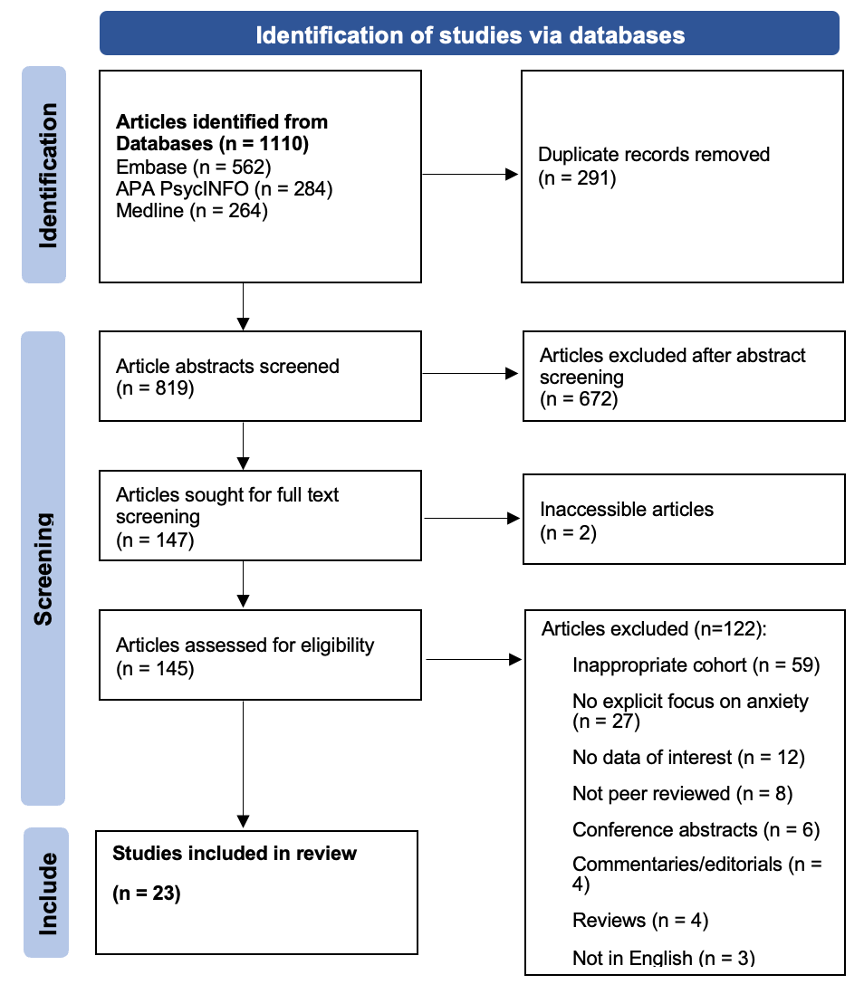
A.5: Research Results

A.6: Study Characteristics Table

| **Title** | **Authors (Year)** | **Country** | **Type of Study** | **Sample Characteristics** | **Exposure Measure** | **Outcome Measure** | **Main Findings** |
| --- | --- | --- | --- | --- | --- | --- | --- |
| Social comparison and state-trait dynamics: Viewing image-conscious Instagram accounts affects college students' mood and anxiety [37]. | Kohler, Turner and Webster (2021) | USA | Randomised control trial | 81 university students, Female: 65.4%,  Male:  34.6%,  Mean age = 19.07 (SD=1.56),  Age range =  18-30 | Instagram | State Anxiety    Scale:  GAD-2 (measured pre- and post-Instagram scrolling) | Marginal increase in state anxiety in Experimental group, exposed to 10 mins of appearance focused Instagram accounts (beauty, fitness, model, compared to control group, who were exposed to home décor, nature and food accounts  (r = 0.21, p<.001) |
| Predicting Facebook addiction and state anxiety without Facebook by gender, trait anxiety, Facebook intensity, and different Facebook activities [40] | Xie and Karan (2019) | USA | Cross-Sectional Study | 526 Students, Females: 59%, Males: 41%, Mean Age (years): 24.21 (SD = 5.92) | Facebook  Scale:  Facebook Intensity Scale  And  Bergen Facebook Addiction Scale | Trait Anxiety  Scale:  State-Trait Anxiety Inventory | People with higher level of Facebook addiction reported higher level of state anxiety without Facebook (β = 0.291, p < .001, 95% CI [0.202, 0.338]). |
| Correlation between psychological factors, academic performance and social media addiction: model-based testing [42] | Malak, Shuhaiber and Al-Amer (2021) | Jordan | Cross-Sectional Study | 10 university students, Females: 68.6%,  Males: 31.4%, Age (years): Mean: 21.38 (SD = 2.12) | Social Media Addiction  Scale:  Social Media Addiction Scale | Anxiety  Scale:  Symptom Checklist 25 (The anxiety subscale, 10 items) | Social media addiction is significantly and positively associated with anxiety (r = 0.347, p<0.01). PLS algorithm showed that the influence pathway of social media addiction on anxiety was statistically significant (0.388, p<0.001). |
| Social media addiction and burnout: The mediating roles of envy and social media use anxiety [38] | Liu C and Ma J (2018) | China | Cross-Sectional Study | 519 university students, Females: 58.4%,  Males: 41.6%, Age (years): Mean: Men (19.42 +/- 1.49), Female (18.81 +/- 1.10) | Social Media Addiction  Scale:  Self-developed scale with Likert scale questions based on the most popular social medias in China. | Social Anxiety  Scale:  Self-developed scale (21 items, categorised into 4 subscales: shared content anxiety, privacy concern-related anxiety, interaction anxiety, and self-evaluation anxiety) | Social media addiction and anxiety were positively correlated (r = 0.556, p <0.01)   Bayesian SEM analysis showed that social media addiction is a significant predictor of social media anxiety (mean = 0.2888, 95% CI, [0.2255, 0.3515]).   Additionally, social media use anxiety was found to be predicted by social media induced envy and this relationship was significant. |
| Problematic Smartphone and Social Media Use Among Bangladeshi College and University Students Amid COVID-19: The Role of Psychological Well-Being and Pandemic Related Factors [43] | Islam et al (2021) | Bangladesh | Cross-Sectional Study | 5511 college and university students (22.9% College, 77.1% Univ), Females: 41.1%, Males: 58.9%, Age (years):21.2 (SD= 1.7) Age range - 18-25 | Social Media Addiction  Scale:  Bergen Social Media Addiction Scale | Anxiety  Scale: Generalised Anxiety Disorder-7 Scale – GAD-7 | Problematic social media usage was positively associated with anxiety (β = 0.18, p<0.001) |
| Problematic Social Media Usage and Anxiety Among University Students During the COVID-19 Pandemic: The Mediating Role of Psychological Capital and the Moderating Role of Academic Burnout [44] | Jiang (2021) | China | Cross-Sectional Study | 3123 undergraduates Females: 50.2% Males: 49.8% | Problematic Social Media Usage  Scale: Problematic Mobile Social Media Usage Assessment Questionnaire | Anxiety  Scale:  Generalised Anxiety Disorder-7 Scale – GAD-7 | Problematic social media usage was positively correlated with anxiety levels: Group 1 (Academic performance affected by covid-19): β = 0.39, p<0.001  Group 2: (Academic performance unaffected by covid-19): β = 0.32, p <0.001 This means that regardless of academic performance, anxiety was affected by problematic social media use |
| Longitudinal effects of social media experiences on depression and anxiety in LGB+ and heterosexual young adults [45] | Pellicane et al (2020) | USA | Longitudinal Observational  Study | 382 college students Females: 84.1% Males: 15.9% Mean age=19.87 | Social Media Experiences Questionnaire  (Only the ‘Experiences of Acceptance and Experiences of Hostility’ subsection was used) | State anxiety  Scale: State anxiety subscale of the ‘State-Trait Anxiety Inventory’ | Higher levels of acceptance on social media results in lower levels of anxiety in the LGBTQ+ community measured at three points in time.  Correlation between social media acceptance and anxiety symptoms was non-significant in all surveys (survey 1 = -0.04, survey 2 = -0.12, survey 3 = -0.03) |
| The effects of self-disclaimer Instagram captions on young women's mood and body image: The moderating effect of participants' own photo manipulation practices [36]. | McComb et al (2021) | Canada | Randomised controlled trial | 311 undergraduate students Females 100% Mean Age: 19.13  Age range: 18-25 | Instagram  Exposure to photos of thin idealised women | Anxiety  Scale: Self-developed questionnaire using visual analogue scale | Results indicated that there was a main effect of time in which anxiety decreased after exposure to the thin ideal images (p < 0.01) |
| Assessing the impact of Social Media Use on everyday emotion in health crises: A study of international students in China during Covid-19 [46] | Larnyo et al (2015) | China | Cross-Sectional | 474 university students, Female: 41.4%, Male: 58.6%Median age = 24 (SD=6.21) | Social Media Experiences  Scale: Social Media Experiences Questionnaire (constructed from a Facebook addiction scale) | Anxiety.  Scale: GAD-7 scale | Significant positive impact of social media use on everyday emotions (anxiety and depression) of international students (r = 0.34, p < 0.05).  Self-rated anxiety and depression associated with social media use among international students were generally mild (n = 249, 52.50% and n = 350, 73.80%, respectively). |
| Associations Between Instagram Addiction, Academic Performance, Social Anxiety, Depression, and Life Satisfaction Among University Students [47] | Foroughi et al (2021) | Malaysia | Cross-sectional | 364 university student, Female: 51.5%, Males: 48.9%, Range = 19-26 | Instagram Addiction. Scale:  Bergen Facebook Addiction Scale- BFAS. (The term “Facebook” was replaced with “Instagram” | Social Anxiety.  Scale: Social Avoidance and Distress Scale – SADS (Specific to new people or situations) | Instagram addiction had a statistically significant positive association with social anxiety. (β= 0.406, p <0.05) |
| Social Media Use and Mental Health during the COVID‐19 Pandemic: Moderator Role of Disaster Stressor and Mediator Role of Negative Affect [48] | Zhao and Zhou (2020) | China | Cross-sectional | 512 college students, Female: 62.5%, Male: 37.5%, Mean age = 22.12  (SD=2.47) | Social Media Experience  Scale: Social Media Experiences Questionnaire.  Social Media Usage  Scale: Assessment tool developed by Lin et al | Anxiety  Scale:  GAD-7 scale | Associations of social media use with anxiety was significant (r = 0.10, p < 0.05)  Disaster-related social media consumption was significantly associated with negative mental health (anxiety included under this broad term). (r = 0.20, p <0.001).  Traditional and Online media usages were not statically significantly associated with anxiety (r=0.02, r=0.05 respectively).  Negative affect mediated relationships between social media use and anxiety (b= .41, 95% CI [0.13, 0.68]) |
| Characteristics of social media 'detoxification' in university students [49] | El-Khoury, Haidar, Kanj et al (2020) | Libya | Cross-sectional | 68 university students, Females: 45.6%, Males: 54.4%,  Age (years): All participants were 18+ | Social media detoxification. Scale: Self-developed 23 item questionnaire | Anxiety.  Scale: Self-developed survey- individual experience reported during period of detoxification (participants could choose increased/decreased anxiety) | Most students (N = 42, 62%) denied feeling restless or troubled when prohibited from using social media. Amongst the cohort that underwent social media detoxification (n=27), 16.2% reported decreased anxiety, and 5.8% reported increased anxiety. These results are non-significant. |
| I want you to like me: Extraversion, need for approval, and time on Facebook as predictors of anxiety [31]. | Steers, Quist, Bryan et al (2016) | USA | Cross-sectional | 280 college students  Female: 70% , Male: 23.35%, Mean age = 23.35 (SD= 5.88) | Time spent on Facebook | Trait Anxiety  Scale: Trait anxiety subscale (part of the State-Trait Anxiety Inventory) | Anxiety had a positive relationship with time spent on Facebook (r=0.19, p <0.05). |
| Relationships between Severity of Internet Gaming Disorder, Severity of Problematic Social Media Use, Sleep Quality and Psychological Distress  [50] | Wong, Mo, Potenza et al (2020) | Hong Kong | Cross-sectional | 300 university students Females: 59.33%, Males: 40.67%, Mean age = 20.89 (SD=1.48) Range = 18-24 | Time spent (hours) on social media, gaming or on smartphone.  General social media addiction Scale: Bergen Social Media Addiction Scale (BSMAS). | Anxiety.  Scale: Anxiety subscale of the Depression Anxiety Stress Scales (DASS-21) | There was a statistically significant positive correlation between social media addiction and anxiety (r=0.344, p<0.001).  The regression coefficient between BSMAS scores and anxiety r= 0.196, p<0.01) |
| The effect of social media exposure on depression and anxiety disorders in facing Covid-19 pandemic [33]. | Widiyanto, Fajriah and Atmojo et al (2020) | Indonesia | Cross-sectional | 430 participants, Female: 74.88%, Males: 25.12% | Social media exposure. Scale: Own classification measured by exposure to news and information about COVID-19 on social media | Anxiety.  Scale: GAD-7 | Higher frequency of social media exposure was insignificantly positively associated with anxiety disorders after controlling for all covariates. |
| Higher emotional investment in social media is related to anxiety and depression in university students [32]. | Alsunni and Latif (2020) | Saudi Arabia | Cross-sectional | 893 University students Females: 51.1%, Males: 48.9%, Mean age = 23.91 (SD=2.17) | Volume and Frequency of use for 6 social media platforms were measured.  Night-time Social Media Usage.  Scale: Woods & Scott Questionnaire  Emotional investment in social media  Scale:  Social Integration and Emotional Connection subscale of the Social Media Use Integration Scale | Anxiety  Scale: Hospital Anxiety and Depression Scale (HADS) | Statistically significant positive correlation between emotional investment in social media and anxiety (r = 0.71, p <0.001).  A per unit increase in emotional investment meant that participants were 1.76 times more likely to be anxious.  Night-time specific and overall social media use had no significant effect on being anxious or developing anxiety.  Emotions are more important than time spent on social media when predicting levels of anxiety caused by social media. |
| Do motivations for using Facebook moderate the association between Facebook use and psychological well-being?  [41] | Rae and Lonborg (2015) | US | Cross-sectional | 119 University Students; Female 73.6%; Male 26.4%, Age Range = 18-25 | Quantity of Facebook Use    Scale: 9-Point scale to measure number of Facebook friends;  6-Point scale for time spent per day (Ellison et al, 2007);  Motivations for Facebook Use  Scale:  11 self report items (Raacke and Bonds-Raacke) | Anxiety  Scale: Mental Health Inventory Sub-scales (Veit and Ware, 1983) | Time spent using Facebook predicted higher levels of anxiety, but only among the users that most frequently accessed the site for connection reasons.  Time spent using Facebook was not a significant predictor of anxiety at one SD below the mean of connection purposes (β = -0.20, p = 0.234), but was a significant predictor at one SD above the mean (β = 0.42, p = 0.013). So time spent using Facebook predicted higher anxiety in the most frequent users, but only those that used it for connection purposes. |
| Impact of Social Media on Mental Health among Medical Students of Private Medical College, Sargodha [51] | Ghani et al (2021) | Pakistan | Cross-sectional | 150 undergraduates  Female: 72.7%, Male: 27.3% | WhatsApp use pattern questionnaire (WUPQ) | Anxiety  Scale: Hospital Anxiety and Depression Scale (HADS) | Study found that students with greater WhatsApp dependence suffered from higher levels of anxiety (r = 0.229, p <0.01) |
| A Cross-sectional Study on Patterns of Social Media Chat Usage and its Association with Psychiatric Morbidity among Nursing Students [52] | Hamsa, Singh and Kaur (2020) | India | Cross-sectional | 500 nursing students, Females:  100%, Mean Age (years): 21.05 ( SD = 0.79) | Social media addiction.  Scale: Bergen facebook addiction scale (modified for WhatsApp Usage). | Anxiety.  Scale: Depression Anxiety Stress Scales (DASS) | There was a weak positive correlation between WhatsApp usage and anxiety (r = 0.2, p <0.01). |
| The patterns of problematic social media use (SMU) and their relationship with online flow, life satisfaction, depression, anxiety and stress symptoms in Lithuania and in Germany [34]. | Brailovskaia et al (2021) | Lithuania and Germany | Cross-sectional | Lithuanian sample. 1640 university students, Female: 68.5%, Males: 31.5%. Mean Age (years): 19.09 (SD = 1.05) | Problematic social media use.  Scale: Shortened version of the Bergen Social Media Addiction Scale (BSMAS; Andreassen et al. 2017). 5 point Likert scale for 6 core features of problematic social media use.  Social Media Flow. Scale: Modified version of the 'Facebook Flow Questionnaire' (FFQ; Brailovskaia et al. 2018; Kwak et al. 2014). | Anxiety.  Scale: Depression Anxiety Stress Scales 21 (DASS-21; Lovibond and Lovibond 1995) | The Latent Class Analysis classified the individuals according to levels of problematic SMU characteristics: their levels of problematic SMU characteristics: low-symptom, low-withdrawal, high-withdrawal, and high-symptom. Low-symptom group, compared to all other groups (p < .001), reported the lowest levels of anxiety (βlow-withdrawal = 0.21). The high-symptom group, compared to low-withdrawal and high-withdrawal groups (p < .001), reported higher levels anxiety (βlow-withdrawal = −0.29; βhigh-withdrawal = −0.31). |
| Emotion regulation’s relationships with depression, anxiety and stress due to imagined smartphone and social media loss [39] | Elhai et al (2018) | USA | Experimental study | 396 College Student, Female: 74.9%, Males: 25.1%, Mean Age (years): 19.01 (SD= 1.26) | Social media loss imagined for one group, keeping the other a control. | Anxiety.  Scale: Shortened version of the Depression Anxiety Stress Scale (Lovibond and Lovibond, 1995) consisting of 21 items. | The loss of social media was significantly positively associated with anxiety (p < 0.001, η = 0.21). |
| What does media use reveal about personality and mental health? An exploratory investigation among German students [53] | Brailovskaia and Margraf (2018) | Germany | Cross-sectional | 633 Freshman (419 W; 214 M)  Mean age: 21.80 (SD: 5.35; Range: 16-59) | SNSs use was determined using the question: “How often do you use social platforms?”). | Anxiety.  Scale: Depression Anxiety Stress Scales 21 (DASS-21) | SNSs use did not independently predict anxiety and stress symptoms over and above the othervariables.  Twitter use was significantly correlated with anxiety (0.11; p<0.01)  Tumbler use was also significantly associated with anxiety (0.08; p<0.05)  SNSs use showed a very weak but significant result (SNSs use: changes in R2= .004, F(1,618) = 4.551,p = .033; standardized beta = .066, p = .033, 95% CI [.013;.323]), in the model with anxietysymptoms as dependent variable (SNSs use: changes in R2= .000, F(1,618) = .015, p = .903;standardized beta = .004, p = .903, 95% CI [-.131;.148]) |
| No more FOMO: Limiting social media decreases loneliness and depression [35]. | Hunt et al (2018) | USA | Experimental study | 143 undergraduate students  Female: 75.5%, Males: 24.5%. Mean Age (years): NR. Range: NR | Tracking of social media usage using iPhone activity | Anxiety:  Scale: State-Trait Anxiety Inventory (STAI; Speilberger et al., 1970)  FOMO:  Scale: The fear of Missings Out Scale (FoMOs; Pryzbylski, Murayama, DeHaan & Gladwell, 2013) | There was an observed decline in anxiety in the control group t(46) = 3.035, p < 0.004, and experimental, t(65) = 2.477, p < 0.016, groups, where the control group used social media as usual and the experimental group were asked to limit their social media use to 10 minutes per day on Facebook, Snapchat and Instagram. |
