# Supplementary material for: Exploring the Impact of Social Media on Anxiety Among University Students in the United Kingdom: Qualitative Study
Source: JMIR Form Res. 2023 Jun 16;7:e43037. doi: 10.2196/43037 (PMC10337317; doi:10.2196/43037)
Supplement: Multimedia Appendix 2 [file formative_v7i1e43037_app2.docx]

**Multimedia Appendix 2.** Thematic Analysis.

2.1: Factors Decreasing Student Anxiety Levels

| **2^nd^ order** | **Subtheme** | **1^st^ Order** | **Quotes** |
| --- | --- | --- | --- |
| **Social Connections** | Connect with friends and family | Maintain friendships | *I’m a very social person so I like that side of social media. It helps me stay connected with people all around the world, even my cousins who live abroad. I can talk to my friends or family that I haven’t seen for a while. (Student 22)* |
|  |  |  | *Like I said earlier, it's a really nice way to stay in touch with people. And I think that there's probably a lot of friends from when I was younger that I wouldn't be in touch with if I didn't follow them on Instagram. It's such an easy way that doesn't really require any active effort, just to see what people are up to and to post or leave a comment on it. The quick message feature opens up those avenues of communication that is so easy to do. You don't really have an excuse not to. (Student 15)* |
|  |  |  | *And now we're more connected than ever, we get to know what people are up to. Because in the end, where most people tend to be quite sociable, and we're social animals, right? So we want to see what people are up to, we want to talk to them and vice versa. People want to know what you're up to. And it's a good platform for that. (Student 1)* |
|  |  | Build Friendships | *And now I've met like some of my closest most dear friends through it. (Student 7)* |
|  |  |  | *I think reconnecting with people that I haven't met in a long time. I think [Redacted] is a good example. So I lost contact with him for 10 plus years. We found each other through Instagram. So we started messaging again. (Student 12)* |
|  |  | Relieve Loneliness during COVID | *In terms of my mental wellbeing, it can help me stop loneliness and keep in touch with friends and family especially when I’m at university. (Student 27)* |
|  |  |  | *So I remember this was during COVID and we did this thing where everyone would run on Strava then we'd be posting on our Instagram stories how much we’d run for the day. (Student 16)* |
|  |  |  | *So even though you're at home, by yourself, or with just your family, you're actually like, connected with the world out there. And I thought that was really really nice. (Student 6)* |
|  |  | Connect with the family | *I think I place a lot of value on social media because I am using it a lot so I can’t say that it doesn’t give me any value from it. It is good because it helps me stay connected to all my friends and family. That is the main reason why I think social media is beneficial for me. (Student 22)* |
|  | Online Communities and support | Involvement in online communities and networks | *Yeah, I came into this football writing stuff as a novice who didn’t know what they were doing. You interact with some people, one thing leads to another, and you are in a group-chat with all these people who are massive in the football writing industry. It was only through Twitter that I was exposed to all of this, where so many people want to help you. I joined a few months back and these people taught me how to code, giving me a free education on the platform. (Student 21)* |
|  |  |  | *So there was a Ramadan in lockdown, where a lot of people were then doing like the virtual calls and virtual iftars and virtual like, talks and things and it created such a nice community. (Student 6)* |
|  |  |  | *I would say it has impacted it in a positive manner because of the social media community I am in. It depends on the social media community you’re in but I’m fortunate enough to belong in one where everyone seeks to uplift one another, with the work you are producing being praised and promoted. Everyone on it is in a good space. It is a really nice support group to have, to be honest. (Student 21)* |
| **Positive Experience** | Positive Content | Spiritual and religious content | *social media has benefited me a great deal in terms of coming closer to my own faith. I think it is a very personal topic. I follow a lot of religious accounts on social media and they tend to show positive, bitesize clips that really provides me with hope and motivation to continue being strong in your faith. There are also so many motivational quotes and videos that you come across a lot, and I think that really helps you to better yourself. The feeling of hope is further substantiated when you see other people on social media talk about their feelings on our religion or videos that you will connect with. (Student 27)* |
|  |  |  | *Sometimes it has had a positive impact like when I see Christian verses that can be positive and motivating. (Student 25)* |
|  |  |  | *Islamic content for me personally, like, whether it be like small reminders in the form of a video or in the form of like a text quote or whatever, that's what I be more likely to save in my like little saved folder. Kind of grounds me. (Student 6)* |
|  |  | Relatable Content | *It gives me ideas, seeing different things that people are doing and the different places that people go to. Especially with Instagram, seeing nice photos, for example, all of that can be inspiring and I can apply it in my own life. So in terms of creativity, it makes me happy seeing other peoples work and then using that to inspire myself. (Student 17)* |
|  |  |  | *There is a lot of nice content out there. For example, TikTok is full of shows I’m watching right now so there is a relatable aspect for me there. Yesterday, I had my theory lesson so stuff about driving came up and it was useful for me, actually. (Student 23)* |
|  |  | Informational Content | *Also I feel like I do like following people and accounts that I feel will have a more of a positive impacts on me. So I can follow someone that has a healthy living account for example I find that seeing posts like that would encourage me to be better or to go out and go for a run or pick something healthy to do. You can pick and choose obviously which influencers you follow. But I think if you follow influencers that help you and have a good impact on you than I do get positives from that as well. (Student 15)* |
|  |  | Activism - Raise awareness and consolidatory for important issues | *I guess I've learned a lot from people through social media, just different people’s mindsets and different people’s viewpoints. I think specially with certain campaigns, such as BLM for example, that really showed the power of people when they come together. Obviously there's pros and cons to that. I think with the BLM campaign, it was really good because it was towards such an important topic. (Student 14)* |
|  |  |  | *And also having links to raise money for charity. So that's the positives. (Student 16)* |
|  | Positive Validation | Positive Validation in the form of likes comments and messages | *Also, the endorphins that come with posting or the number of likes does have a positive impact on me. (Student 22)* |
|  |  |  | *I also think from a vain perspective, but I think everyone would agree with this, it's nice getting positive feedback from people on stuff that you posed and it’s nice receiving compliments, but also complementing other people on stuff they've posted. (Student 15)* |
|  |  |  | *If I was to post, I will only post what I really like to and what I really want to post. Before, I’d post things to get some reactions out of people. I know this might sound big-headed, but I like getting compliments and interactions. (Student 22)* |
|  |  |  | *Obviously there's positive validation when people like your post and comment. (Student 19)* |
| **Escapism** | Distraction | Distraction from Mental Health | *If there is anything in the real world that affects my anxiety, funnily social media can act as a way of helping me cope with it. It puts my mind in another place; a place where I don’t have to think too much. (Student 22)* |
|  |  |  | *quite a good numbing agent. Hmm, that especially when I had like very severe depression, alongside like anxiety for a bit I felt that social media was a really good distraction to just not think about what what was going on in my head. And so in some senses, it was a good escapism. (Student 4)* |
|  |  |  | *I think the numbing effect was the most important thing. It was something to like, pull me out of my like, state of like despair, into anything that's stimulating. That's why I was like, watching things that were just like, utter trash, like it made no sense. But like, it was something to just distract me. And that's what I needed at that time. So it was beneficial in the like in that distracting sense. (Student 4)* |
|  |  |  | *So when I feel a lot of different emotions, even anxiety, I will go on social media and sometimes it makes it worse and sometimes it makes it better. (Student 10)* |
|  |  | Escapism from reality | *A massive one, I think. I don't think I could live without social media. It's because of the communication aspect. You can be in contact with anyone in the world within just seconds, and just having that sort of access to information. TikTok is fantastic again, because it allows you to escape reality within seconds. (Student 14)* |
|  | Relief | Calming and destressing effects of SM | *It‘s also a nice break in the day so if you had a busy day at work you can just have a quick scroll through. (Student 16)* |
|  |  |  | *Stuff like that can be a pretty ample distraction. So I would say that that's, that's true. Like, sometimes if you're just feeling a bit you know, as you put like, a bit anxious or maybe overwhelmed. Just doing something pretty mundane, can sort of help. So I suppose yeah, social media can also prove as a as a distraction as well. (Student 20)* |

2.2: Factors Increasing Student Anxiety Levels

| **2^nd^ order** | **Subtheme** | **1^st^ Order** | **Quotes** |
| --- | --- | --- | --- |
| **Comparison** | Comparison of Life | Comparison of life | *When people post a lot of content about their lives whilst you’re just at home, not doing anything special, you feel anxious in that you should really be doing something. Or that I am not doing enough. I guess I do feel the pressures of social media at times. (Student 21)* |
|  |  |  | *just looking at the success of others and comparing it to my lack of success really heightened my anxiety and pushed me down further. So, it needs to be explored more. (Student 27)* |
|  |  |  | *Maybe social media has impacted my mental health in a negative way, subconsciously though. You always want to do better and when you see other people flourish on social media, you constantly compare yourself to them and that feeling can often be a little bit overwhelming. (Student 21)* |
|  |  |  | *Sometimes you can be a bit jealous of other people doing cool things or having cool lives. (Student 12)* |
|  |  |  | *So you can actually see who is financially more well off or of a higher social economic class and then you're kind of comparing yourself to these people who have more money than you and are older than you, and therefore will be more successful and accomplished. You then compare and that kind of comparison is just unhealthy. (Student 10)* |
|  |  | Comparison of Likes, Comments & Followers | *I was just looking at everyone else, in terms of my peers maybe influences, seeing how much I don't know, how many followers they had, how many friends they had, how many likes they got, and be paranoid about things like, oh, I don't have people, I don't have friends, because I will be constantly comparing myself to these influences, etc. (Student 2)* |
|  |  |  | *Yeah, I think now that you say, I think I think it's been, it was kind of an anxiety thing more when I was younger a couple of years ago, compared to now I think now it's not that definitely, let's say on Instagram, or something like that, and you you'd be seen, or how many likes people getting or, you know, that sort of comparison thing? I think that did give me a bit of anxiety back in the day. (Student 1)* |
|  | Comparison of Body types | Comparison of Body Types | *I had like an issue with body image. It wasn’t a big thing, but it played on the back of my mind. And with social medias like Instagram, where a big part of it is posting photos of yourself, I could definitely see why it would be a negative. (Student 11)* |
|  |  |  | *I think it comes down to influencers – like they have good body types and things that can make you quite insecure. (Student 25)* |
|  |  |  | *Sometimes it was study-related, but that was very infrequently – but a lot of gym stuff as well, which did exacerbate how inadequate I felt as well. You see all these people your age, who are 10 times bigger than you, they have a better diet – that was tough to take. That sort of content really did make my anxiety worse. (Student 26)* |
|  |  | Comparison exacerbating insecurities | *I think that definitely contributes to kind of the background stress, but I think in terms of the other side of things and kind of sort of the insecurity like low self esteem kind of side of things, like a confidence side of things that they can start probably a lot worse. (Student 5)* |
|  |  |  | *In several ways I can I like, I've seen things on Tik Tok, that people label as insecurities, which literally just shouldn't exist as insecurities. Like unlocking new insecurities. I remember seeing a video that was like, I always check my back profile before I leave, and everyone was like, you've unlocked a new insecurity because I didn't even think to think that. (Student 7)* |
|  |  |  | *And Tik Tok especially goes on an algorithm where like, if you like something once, it's just like, everywhere. And if I like one summer body TikTok, I'm just bombarded by like, beautiful women and like, good for them. But I'm just like, I have spot cream on, I just don't need to see this. (Student 7)* |
| **Fear of missing out (FOMO)** | FOMO | FOMO | *I would not use my phone for even just 2 hours and I would feel like I was missing out on something. This would drive me crazy and add so much unnecessary stress. My phone would be constantly buzzing in my pocket with notifications from the group chat with messages coming through constantly. This would make me feel like I was missing out on something and would drive me to constantly be on my phone. (Student 24)* |
|  |  |  | *You see so many different stories of people doing different things. Sometimes you get that fear of missing out. (Student 11)* |
|  |  |  | *People around me were always on their phones using these apps, so naturally, I would do the same. If I wasn’t on it, it would bring the fear that I felt like I was missing out. This FOMO really kicked in at ages 15-17. (Student 24)* |
|  |  |  | *Maybe when people are doing a lot of things and you're like, ‘oh, I'm missing out on so much’, but not to the extensively. It's just like a fleeting feeling. (Student 19)* |
| **Procrastination** | Procrastination | Procrastination from priorities | *So, because I am a university student if I have a deadline or things to do, like exam preparation, I am not one to just focus on that. I tend to procrastinate an unhealthy amount. Social media takes time away from me completing my uni work, doing my chores or doing things to better myself and, in turn, this increases my anxiety levels. (Student 22)* |
|  |  |  | *Whenever I am, for example, working or being occupied with something, my first impulse is to go on my phone and use social media. It is so easy to consume – just go on your phone and start scrolling. My first impulse is to go on TikTok or YouTube. It is just an instinct. This is especially when I am working at something hard and I come to a mental block, I tend to take the easy option and jump onto social media. I don’t really go on it with any real purpose but I just go on it. (Student 24)* |
|  |  |  | *Yes, when I have a lot of deadlines or when I'm meant to be doing work, it will kind of irk me that I've been using too much social media. (Student 12)* |
|  |  | Time consuming | *I think the main thing is about the time it takes up. So it uses up so much time and I can find myself having been on TikTok for like 2 hours. And then I just wouldn't have done any of the work I wanted to do and you just lose track of time. So I think that's the worst thing. (Student 18)* |
|  |  |  | *Sometimes social media can be a good thing, and sometimes it can make me feel worse. I'd say most times I do take positive things from it, but then I'm kind of conscious that if I spend too much time on it then I won't feel good afterwards and I'll regret spending too long on a certain social media when I should have been doing something else. (Student 15)* |
|  |  |  | *But afterwards I'll be like I just wasted how ever much time on there and I could have been doing something a bit more productive. (Student 12)* |
| **Stress** | Feeling overwhelmed | Feeling overwhelmed by large number of notifications and messages | *So I feel like with WhatsApp, there's kind of a chronic stress and background, especially when you have like later messages and stuff coming through and you can kind of feel obliged to kind of just continually be online and be active. (Student 5)* |
|  |  |  | *just generally speaking ive got 6/7 different conversations going on anyway. So, all of that combined becomes very hard to manage. There have been moments in time where ive become overwhelmed and anxious and not wanting to go on my phone at all. (Student 9)* |
|  |  |  | *I think it facilitates hyper connectivity, to an extent to which you should not interact with that many people a day, you should not be seeing that many people on a screen, like a day and you should not be hearing that many opinions today. I think it just really sends your brain in overdrive mode. Student 7)* |
|  | Overthinking online interactions | Overthinking online interactions | *I know it is sounds bad, but there have been occasions where I would request to follow people that I thought were my friend and they wouldn’t accept back. It is a superficial thing, but you can really be impacted by it. There is so much overthinking with it; thinking that maybe this person didn’t like me. Or perhaps you message someone, and you see that they are online, but they do not message you. Again, sometimes I look at this and think ‘Ok, does this person not have enough time for me?’. This was definitely in my younger years at uni, nowadays this isn’t so much the case. (Student 27)* |
|  |  |  | *you might be talking to someone that you have some hope for it going somewhere, and then suddenly they stop replying. So that’s an interaction on social media and because you’re not held accountable for that interaction it gives you anxiety. (Student 27)* |
|  |  |  | *I mentioned that I only post on my social media that only has my close friends on it now, but even if I do post I’m constantly thinking about everything I post. I nit-pick at the small bits basically. I would question if there were any typos on the post, something wrong in the background or is my appearance only looking nice to me. I’m really picky about it and I feel like that has increased my anxiety for sure. (Student 23)* |
| **Negative Experiences** | Disturbing & Harmful Content | Distressing News | *Sometimes, depending what kind of content is on there, and when something is happened or if I'm having a bad day, it can exacerbate those emotions because I feel that if you're already in a vulnerable position, going on social media doesn't make it better. It just kind of makes it worse. (Student 17)* |
|  |  |  | *With Twitter, it's the same thing, but there's a lot more news that goes round on Twitter, so you see a lot of really sad things as well. So Twitter is a mix of happy and just really depressing. But that's very much reactive, so it's based on what I will see, but the app itself won't invoke a certain emotion. (Student 18)* |
|  |  |  | *I'd feel like awful after watching it, because I just felt like a constant state of anxiety that like, there was such a disparity between a certain set of people who's like, not understanding me. And so like, I think that was probably the worst part was being exposed to videos that have opinions that I just couldn't really wrap my head around. (Student 4)* |
|  |  | Self-harm content | *I think it is scary to think that people can follow pages and accounts that may encourage them to self harm or to commit suicide or to restrict eating or things like this. And I think that I think that a lot of medical professionals and people will just be shocked at kind of how prevalent they are. (Student 15)* |
|  | Judgement by others | Judgement from posting | *I think it mostly lies in the fear of not knowing how it will be perceived by others. I don't think it's an innate thing. I think it's not knowing how other people will react to it or whether it will bang. (Student 18)* |
|  |  |  | *So I would worry more about what other people think of the posts or tweets or worry about the interactions it would get. (Student 3)* |
|  |  |  | *When I tweet for example, people that I don’t know interact with me in a negative way, without any reason which is part of maintaining a public persona on a social media platform. It’s when this happens where I feel like social media can become toxic. (Student 21)* |
|  |  | Judgement for number of likes | *I would have to think twice about keeping pictures up that didn’t receive as many likes as other posts of mine. I would ask myself if there was anything wrong with my posts. Especially because other people can see how many people engaged with your posts through likes and comments, it is embarrassing if you do not hit a certain number. It makes you feel less valued and less appreciated. (Student 27)* |
|  |  |  | *When I was younger, if I was to post and not receive as many likes I would be impacted by it. I wouldn’t understand why I was receiving less interactions with this. This would annoy me, but now as I have got more mature, I don’t think it affects me now. I just post whatever I want to post now. You could say that I place less value on likes and comments now. (Student 22)* |
|  | Cyberbullying | Cyberbullying | *There were a few people who used to DM (direct-message) me. They would talk about my appearance, the way I look and because I am quite self-conscious about that, they would use it to target me and harass me. So that ultimately led to me blocking these people on Instagram. (Student 23)* |
|  |  |  | *I have also seen evidence of bullying on Facebook and Instagram, particularly, when I was in high school. It wasn’t directed towards me, but people I know. So, obviously I was angered and incensed by that. But I haven’t seen anything directed towards me which has upset me. But I have seen things directed at others which has angered me. (Student 26)* |
|  |  |  | *Before, there was a time where it got to me in a negative way but I managed to control that. I used to use Instagram quite a lot, and I would receive lots of negative comments on that so I stopped using that platform now. I don’t use it all that often now, except to like other peoples’ posts. (Student 23)* |
